# Developing a Treatment Planning Software Based on TG-43U1 Formalism for Cs-137 LDR Brachytherapy

**DOI:** 10.5812/ircmj.4938

**Published:** 2013-08-05

**Authors:** Sedigheh Sina, Reza Faghihi, Ali Soleimani Meigooni, Zahra Siavashpour, Mohammad Amin Mosleh-Shirazi

**Affiliations:** 1 Radiation Research Center, Shiraz University, Shiraz, IR Iran; 2 Department of Nuclear Engineering, Shiraz University, Shiraz, IR Iran; 3 Comprehensive Cancer Center of Nevada, Las Vegas, Nevada, USA; 4 Department of Radiation Medicine, Shahid Beheshti University, Tehran, IR Iran; 5 Center for Research in Medical Physics and Biomedical Engineering, Shiraz University of Medical Sciences, Shiraz, IR Iran; 6 Department of Radiotherapy and Oncology, Shiraz University of Medical Sciences, Shiraz, IR Iran

**Keywords:** Monte Carlo Method, Intracavitary Brachytherapy, PLATO

## Abstract

**Background:**

The old Treatment Planning Systems (TPSs) used for intracavitary brachytherapy with Cs-137 Selectron source utilize traditional dose calculation methods, considering each source as a point source. Using such methods introduces significant errors in dose estimation. As of 1995, TG-43 is used as the main dose calculation formalism in treatment TPSs.

**Objectives:**

The purpose of this study is to design and establish a treatment planning software for Cs-137 Solectron brachytherapy source, based on TG-43U1 formalism by applying the effects of the applicator and dummy spacers.

**Materials and Methods:**

Two softwares used for treatment planning of Cs-137 sources in Iran (STPS and PLATO), are based on old formalisms. The purpose of this work is to establish and develop a TPS for Selectron source based on TG-43 formalism. In this planning system, the dosimetry parameters of each pellet in different places inside applicators were obtained by MCNP4c code. Then the dose distribution around every combination of active and inactive pellets was obtained by summing the doses. The accuracy of this algorithm was checked by comparing its results for special combination of active and inactive pellets with MC simulations. Finally, the uncertainty of old dose calculation formalism was investigated by comparing the results of STPS and PLATO softwares with those obtained by the new algorithm.

**Results:**

For a typical arrangement of 10 active pellets in the applicator, the percentage difference between doses obtained by the new algorithm at 1cm distance from the tip of the applicator and those obtained by old formalisms is about 30%, while the difference between the results of MCNP and the new algorithm is less than 5%.

**Conclusions:**

According to the results, the old dosimetry formalisms, overestimate the dose especially towards the applicator’s tip. While the TG-43U1 based software perform the calculations more accurately.

## 1. Background

The treatment planning systems used for Selectron Cs-137 low dose rate brachytherapy source usually use traditional method of dose calculation, which requires the exposure rate constant and considers each pellet as a point source by applying the correction factors corresponding to photon attenuation and scattering in water. Such algorithms use the superposition method to calculate the dose distribution around every combination of active sources, not accounting for source to source differences in encapsulation or internal construction and the applicator and spacers’ effects. Many investigations have been performed previously for estimating the effect of ignoring the attenuation and scattering due to the applicators and inactive spacers for different brachytherapy sources (1-4). The significant effects of high-Z materials in source covers, spacers and applicator structures have lead the scientists to establish new treatment planning techniques for clinical applications. A new technique was developed by Rivards et al. to implement MC-based brachytherapy dose distribution in the conventional TPS (5). The presence of inactive spacers and different kinds of applicators would have more photon attenuating effects than the phantom material. Therefore not accounting such components would cause a dose overestimation.

In 1995, new dose calculation formalism was introduced by AAPM, known as TG-43, to be used in brachytherapy dosimetry not having the defects of the old dosimetry methods using exposure rate constant. In this formalism and the updated version of it (TG-43U1), the dosimetry parameters of each brachytherapy source should be determined both experimentally and theoretically in a water phantom, and then such parameters are used for obtaining the dose distribution around the source (6). This formalism is used for dosimetry of a single brachytherapy source and the inter source effects and the applicator effects are not considered.

## 2. Objectives

The purpose of this study is to design and establish a treatment planning software for Cs-137 Solectron brachytherapy source, based on TG-43U1 formalism by applying the effects of the applicator and dummy spacers.

## 3. Materials and Methods

### 3.1. Cs-137 Source and Treatment Planning Systems

The Selectron remote after loading LDR Cs-137 unit is composed of active and inactive spherical pellets used in different configurations to deliver the desired dose to the tumor tissue (2, 7-11). This system and its different applicators (i.e. vaginal cylindrical applicators and tandem- ovoids) are widely used in Iran for treatment of vaginal and cervical cancer. Two commercially available planning softwares are used for treatment of patients in hospitals of Iran, PLATO (Version UPS: 11.3), and STPS which is a homemade software (12). Both softwares use similar dose calculation formalism, considering the spherical pellets as point sources. PLATO and STPS softwares calculate the exposure at different distances from the source using the exposure rate constant of Cs-137, the activity of the source, and the inverse square law. Finally, the dose is estimated using Rontgen to Rad conversion factor and applying the absorption and scattering correction factors for the phantom, while ignoring self-absorption in the pellet source and the attenuation of photons in the applicator. The dose distribution around different combinations of active spherical sources is estimated by simple superposition. Such algorithms have been replaced by TG-43 dose calculation formalism since the publication of Task-Group 43 of AAPM. New treatment planning systems are based on this algorithm (6, 13-15).

### 3.2. TG-43U1 Dose Calculation Formalism

In year 1995, Task Group No. 43 of the American Association of Physicists in Medicine (AAPM) was published introducing a new formalism for dose calculation in brachytherapy (6). The first update of the protocol (TG-43U1) was presented in 2004 (14). According to this protocol (TG-43U1), the dose distribution around a sealed brachytherapy source can be determined using the following formalism:

*D(r,ϴ) = SkɅ × G (r,ϴ)/G (r,ϴ) × g(r) × F (r,ϴ)x (r,ϴ)/Gx (r0,ϴ0) × g(r) × F (r,ϴ)x(r) × F (r,ϴ)*

Where Λ is the dose rate constant, g_x_(r) is the radial dose function, F(r, θ) is the 2D anisotropy function and G_x_(r, θ) is the geometry function, and S_k_ is the air kerma strength. The above quantities are defined and discussed in detail in the original TG-43 and the revised TG-43U1 (6, 14).

### 3.3. Monte Carlo Calculation

General-purpose Monte Carlo N Particle code (MCNP version 4c) is used in this study for investigation of TG-43 dosimetry parameters of Cs-137 spherical pellets. The DLC-200 library of photon interaction cross section was used in MC simulations performed in this study. For this purpose, a single Cs-137 pellet source was considered inside the applicator assuming all other pellets as inactive spacers. The active pellet source has been defines as isotropic spherical source emitting the photons of 662 keV. The values of dose at different distances from the source were estimated by dividing the spherical phantom (r = 30cm) into fine cubical lattice with dimension of 1.25×1.25×1.25 mm3 to ensure high resolution sampling in high gradient regions. Tally *F8 was used for scoring the absorbed dose around each source. Coupled photon-electron transport (Mode PE) was used in these simulations. The dose distribution around each pellet was used for calculation of TG-43U1 parameters of Cs-137 source. The TG-43 parameters of pellets were obtained when it was located at different possible positions inside the applicator. All components of the applicator along with the dummy pellets and the active one were simulated using MCNP4C to obtain the TG-43 parameters of each pellet by considering the attenuation and scattering of photons inside such components. In this way we can take the inter-source effects and the applicator effects into account in our dose calculations. The material compositions, densities, and geometry of the active sources, dummy pellets, applicator, and phantoms used for Monte Carlo simulations in this study are shown in Table 1. 

Tally type F6 was used to score the air-kerma rate at different distances (0.5 to 150 cm) from the source center in a 4m radius spherical air phantom, and then the air kerma strength of the source (Sk) was scored by multiplying the simulation results and the distances d squared. The energy cutoff δ = 10 keV was considered in calculation of Sk. 109 histories were considered in our simulations to ensure the uncertainty of dosimetry results and Sk results are acceptable according to the recommendations of TG-43U1 formalism (14).

**Table1.** Mass Density, Composition, and Geometry of the Materials Used in the Brachytherapy Source, Spacers, and Phantom Media

Component	Geometry	Material	Size	Density (g/cm^3^)	Material Composition	Reference
**Source**						
Active core	sphere	Pollucite	Radius = 0.75 mm	2.9	Si (26.18%), Ti (3.00%), Al (1.59%), B (3.73%), Mg (1.21%), Ca (2.86%), Na (12.61%), Cs (0.94%), O (47.89%)	(7, 8)
Source cover	Spherical shell	Stainless steel	Shell thickness = 0.5 mm	7.8	C (0.026%), Mn (1.4%), Si (0.42%), P (0.019%), S (0.003%), Cr (16.8%), Mo (2.11%), Ni (11.01%), Fe (68.21%)	(7, 8)
**Dummy pellets**						
Inactive pellets	sphere	Stainless steel	Radius = 0.75 mm	7.8	C (0.026%), Mn (1.4%), Si (0.42%), P (0.019%), S (0.003%), Cr (16.8%), Mo (2.11%), Ni (11.01%), Fe (68.21%)	(7, 8)
**Phantoms**						
Dosimetry phantom	sphere	water	Radius = 30 cm	1	H (11%), O(88%)	
Sk phantom	sphere	air	Radius = 8 m	0.001	C (0.0124%), N (75.5268%), O (23.1781%), Argon (1.2827%)	(14)
**Vaginal applicator**						
External layer	Cylindrical shell	Stainless steel	Shell thickness = 0.5 mm	7.8	C (0.026%), Mn (1.4%), Si(0.42%), P (0.019%), S (0.003%), Cr (16.8%), Mo (2.11%), Ni (11.01%), Fe (68.21%)	(7)
Air layer	Cylindrical shell	air	Shell thickness = 0.5 mm	0.001	C (0.0124%), N (75.5268%), O (23.1781%), Ar (1.2827%)	(7)
Internal layer (source carrier)	Cylindrical shell	polyethylene	Shell thickness = 0.5 mm	0.93	H (33.33%) C (66.67%)	(7)

### 3.4. Developing a Software Based on TG-43U1 Dose Calculation Formalism

**Figure 1.**
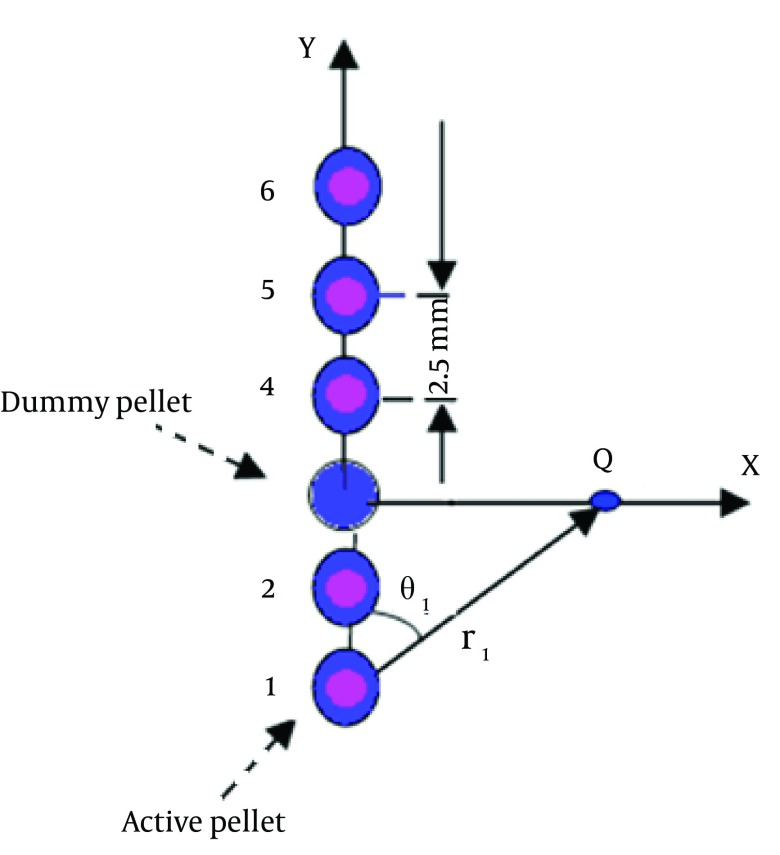
A Certain Configuration Consisting of Five Active Pellets

Once the TG-43U1 parameters of each source at different places inside the applicator are obtained, dose distribution around each configuration of pellets can be calculated using the simple superposition. It should be mentioned that the coordinate center in simulation of each source in the applicator was considered the center of that spherical pellet. Figure 1, shows a certain configuration consisting of six pellets (5 active sources and one inactive pellet). The dose distribution around this combination of active sources can be obtained by adding up the dose around each single pellet calculated using the TG-43 formalism according to equations 2 and 3. 

D_i_ = Dose from source # I to *point Q = Λ. S*_*k*_*. G*_*p*_* (r*_*i*_*, ϴ*_*i*_*). g(r). F (r*_*i*_*, ϴ*_*i*_*)/G*_*p*_* (r*_*0*_*, ϴ*_*0*_*)*

**Figure:**
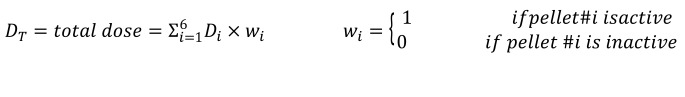


In which ri is the distance between ith pellet source and measurement point (Q), and θ_i_ is the angle between the longitudinal axis of the applicator and the line between the ith pellet source and point Q. The parameters Gp(r_i_,θ_i_) and Gp(r_0_,θ_0_) are the point source approximation of geometry function (inverse square law). Using this method, the attenuation of the source particles in the applicator and other pellets is also taken into account, and the error of dose calculation is minimized.

### 3.5. Verification of the Algorithm

To verify the accuracy of our TG-43U1 based algorithm, the results of this algorithm for a standard configuration of 10 active pellets were compared with the results of direct MCNP4c simulations, and the results of PLATO and STPS software.

## 4. Results

Tabulated dosimetry parameters of each single pellet

As it was explained in the previous section, the TG-43 dosimetry parameters of the single spherical Cs-137 pellet in different positions inside the applicator are needed for use as the input data of the new formalism (using superposition method). Tables 2, and 3 show the values of g(r), and F(r,θ) of the pellet when located in the first possible position inside the applicator. Other tabulated data are also provided to be used as the input of the formalism. 

**Table2.** The Values of g(r) at Different Distances From the Source

r(cm)	1	2	3	4	5	6	7	8	9	10
**g(r)**	1.000	0.998	0.985	0.971	0.947	0.934	0.917	0.906	0.902	0.865

**Table3.** The Anisotropy Function of the First Pellet at Several Angles and Distances

	3 cm	5 cm	7 cm
**15°**	0.903	0.921	0.920
**30°**	0.956	0.961	0.979
**45°**	1	0.996	0.997
**60°**	0.999	0.992	0.995
**75°**	1.003	1.005	1.002
**90°**	1	1	1
**105°**	1.010	1.007	1.010
**120°**	1.003	1.006	1.003
**135°**	0.948	0.988	0.993
**150°**	0.944	0.953	0.958
**165°**	0.902	0.939	0.938

### 4.1. Comparison of the Results of MC Simulations With the Results of Point Source Approximation

**Figure 2.**
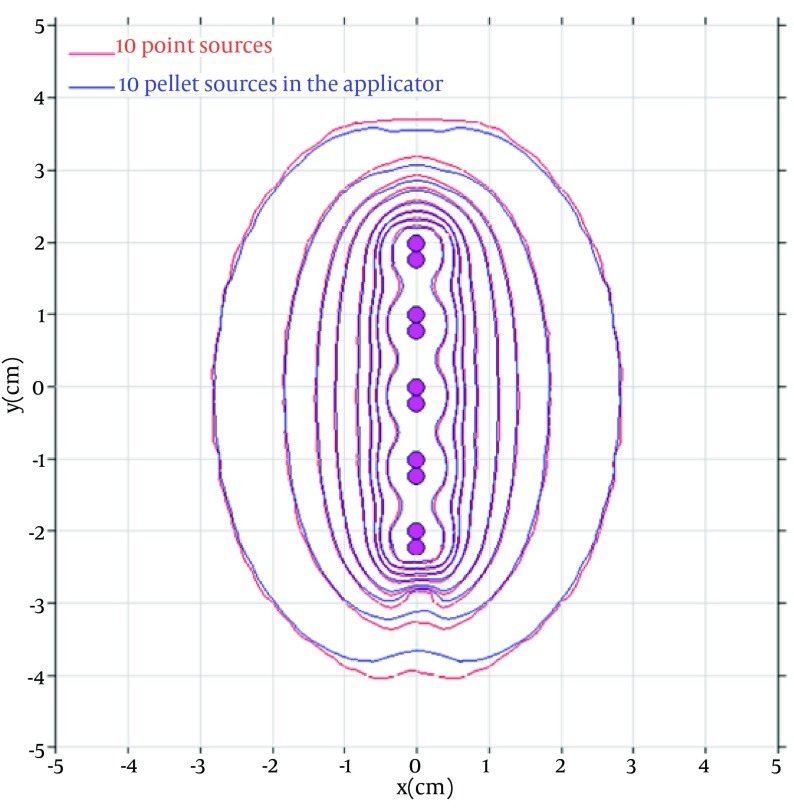
Comparison of the Isodose Curves Around a Standard Configuration of 10 Active Sources in Presence of the Applicator and Dummy Pellets (Blue Lines), With the Dose Distribution Around 10 Point Sources (Red Lines)

The softwares which are currently used for Cs-137 treatment planning, consider each Cs-137 pellet as a point source, and thus no anisotropy exists around the sources. In this study, complete configurations consisting the active source and other dummy pellets inside the applicator were simulated in order to take into account the anisotropy around a single active pellet. The value of anisotropy functions F(r, θ) around a single pellet equals to 1 at all angles and all distances, but if we put this single source inside the applicator, the F(r,θ) values will be different. The values of anisotropy functions for several distances are compared in Table 3. The amount of anisotropy is more pronounced when the number of sources increases. This fact is shown in Figure 2, which compares the dose distribution around a combination of 10 active sources in presence of the applicator and dummy pellets with the dose distribution around ten point sources without the applicator and other pellets. 

As is obvious from Figures 2, the applicator and inactive pellets would have a significant effect on the dose distribution around the Cs-137 pellets. Therefore, we developed a new formalism to be used for dose calculation around Selectron brachytherapy source (see equations 2 and 3). 

### 4.2. Verification of the new algorithm

#### 4.2.1. Comparison of the Results of TG-43U1 Based Algorithm With the Results of Monte Carlo Simulations

**Figure 3.**
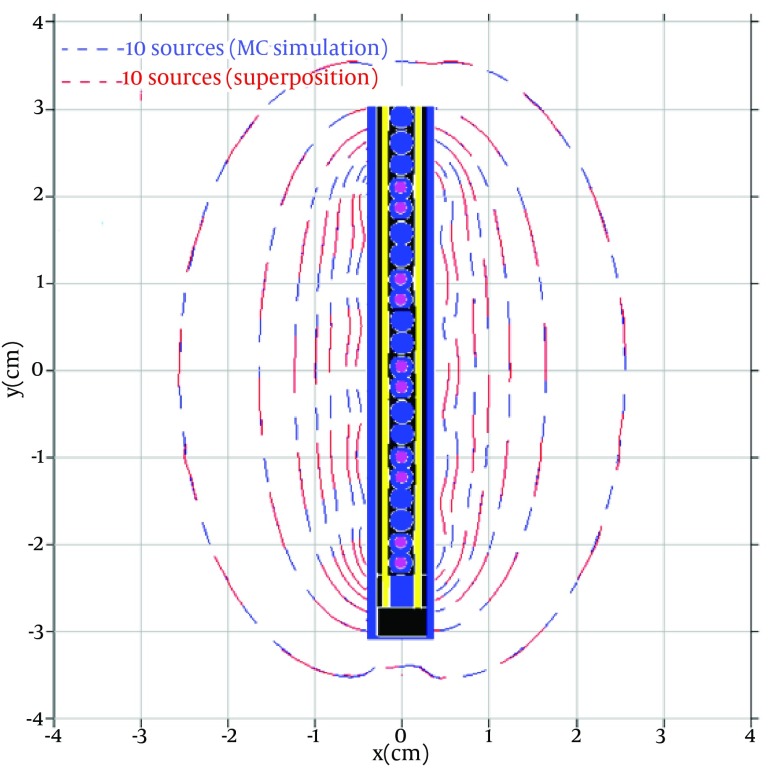
A Comparison Between the Isodose Curves Obtained by Direct MC Simulation (Blue Lines) and the New Software (Red Lines)

To verify the new algorithm using superposition method proposed in this study, the dose distribution around a standard combination of 10 active sources inside the applicator calculated by this method was compared by the dose distribution calculated by directly performing the Monte Carlo simulation of ten active sources (see Figure 3). 

As it is obvious from the figure, the MC results are in great agreement with the results of the TG-43 based software.

#### 4.2.2. Comparison of PLATO, STPS, and the New Algorithm

**Figure 4.**
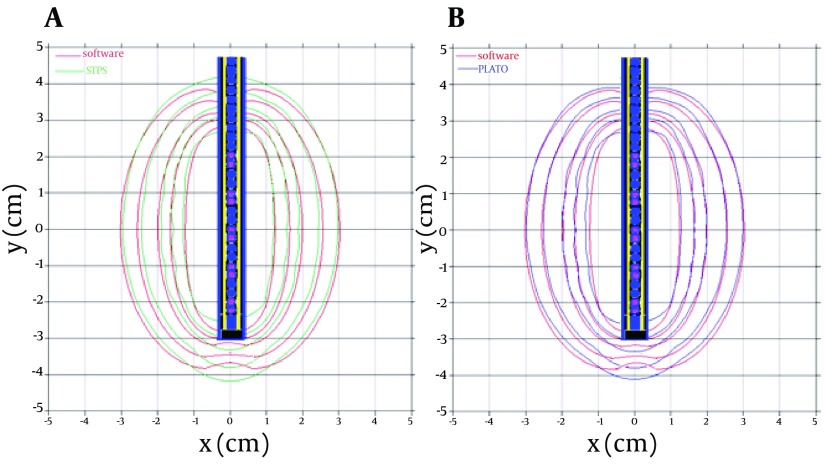
The Dose Distribution Around a Typical Configuration of Active Sources, Obtained by the New Algorithm (Red Lines), STPS (Green Lines), and PLATO (Blue Lines)

Finally the results obtained by the new method were compared by the results of the two treatment planning systems (STPS and PLATO). The percentage difference between the dose obtained by the developed algorithm and STPS software is highest at the tip of the applicator, for example, the percentage of difference between the dose values obtained by the new algorithm at 1cm distance from the tip of the applicator and those obtained by STPS software is about 30% for a typical configuration consisting of 10 active pellets inside the cylindrical applicator. A comparison of the dose distribution around the pellets obtained by TG-43 based algorithm and the results of PLATO and STPS are shown in Figures 4a and 4b. As it is obvious from the Figures, the STPS software would also cause an overestimation of about 30% at the tip of the applicator, while the maximum difference between the results of new algorirhm and MCNP4c simulations is 4% for the point located at r=10cm and θ=165. The MC-based dosimetry parameters implemented in the conventional TPS has improved the dosimetric agreement compared with the direct Monte Carlo dosimetry. The dose calculation improvements obtained in this study are in close agreement with the results obtained by the new MC-based approach developed by Rivards et al for three kinds of applicators and CS-137, I-125, and Pd-103 brachytherapy sources ( 5 ). 

## 5. Discussion

The old treatment planning systems which are currently in use for Cs-137 Selectron source, use the exposure parameters by applying the correction factors for absorption and scatter effects of photons in water. Such algorithms which are used in some treatment planning softwares such as PLATO and STPS consider the spherical active pellets as point sources and do not consider the shielding effects of the source encapsulation and the shielding effects of the applicator and spacers. The new treatment planning systems use the AAPM TG-43 as the reference dose calculation formalism. In this study, a new treatment planning system is developed based on TG-43U1 dosimetry formalism, by considering the attenuation of the radiation in the applicators and the inactive pellets. The results of this study show that the new dosimetry algorithm would improve the dose calculation significantly, especially along the tip of the applicators. The new dosimetry software based on TG-43U1, can replace the old treatment planning systems such as PLATO and STPS for reducing the errors in dose calculations.
